# *HDAC4*, a prognostic and chromosomal instability marker, refines the predictive value of *MGMT* promoter methylation

**DOI:** 10.1007/s11060-014-1709-6

**Published:** 2015-01-04

**Authors:** Wen Cheng, Mingyang Li, Jinquan Cai, Kuanyu Wang, Chuanbao Zhang, Zhaoshi Bao, Yanwei Liu, Anhua Wu

**Affiliations:** 1 Department of Neurosurgery, The First Hospital of China Medical University, Nanjing Street 155, Heping District, Shenyang, 110001 China; 2 Beijing Neurosurgical Institute, Capital Medical University, Beijing, China; 3 Department of Neurosurgery, Beijing Tiantan Hospital, Capital Medical University, Beijing, China; 4 Department of Neurosurgery, The Second Affiliated Hospital of Harbin Medical University, Harbin, China; 5 Department of Neurosurgery, The First Affiliated Hospital of Dalian Medical University, Dalian, China; 6 Chinese Glioma Cooperative Group (CGCG), Beijing, China

**Keywords:** Glioma, HDAC4, Chromosomal instability, MGMT promoter methylation, Prognosis

## Abstract

Chromosomal instability is a hallmark of human cancers and is closely linked to tumorigenesis. The prognostic value of molecular signatures of chromosomal instability (CIN) has been validated in various cancers. However, few studies have examined the relationship between CIN and glioma. Histone deacetylases (HDACs) regulate chromosome structure and are linked to the loss of genomic integrity in cancer cells. In this study, the prognostic value of *HDAC4* expression and its association with markers of CIN were investigated by analyzing data from our own and four other large sample databases. The results showed that *HDAC4* expression is downregulated in high- as compared to low-grade glioma and is associated with a favorable clinical outcome. *HDAC4* expression and CIN were closely related in glioma from both functional and statistical standpoints. Moreover, the predictive value of the *O*-*6*-*methylguanine*-*DNA methyltransferase* (*MGMT*) promoter methylation status—a widely used glioma marker—was refined by *HDAC4* expression level, which was significantly related to CIN in our study. In conclusion, we propose that *HDAC4* expression, a prognostic and CIN marker, enhances the predictive value of *MGMT* promoter methylation status for identifying patients who will most benefit from radiochemotherapy.

**Electronic supplementary material:**

The online version of this article (doi:10.1007/s11060-014-1709-6) contains supplementary material, which is available to authorized users.

## Introduction

Glioma is the most common type of primary central nervous system (CNS) tumor and a leading cause of tumor-related mortality. Despite major advances in therapy over the past decades, the clinical outcome for most patients remains poor. This is especially true for glioblastoma (GBM), the most malignant grade of glioma, which has a median survival of 14.6 months and a 2-year survival rate of 5–10 % even after aggressive therapy [1]. As a major form of genomic instability, chromosomal instability (CIN) is a critical event in early stages of tumorigenesis and, when compounded, leads to the transformation of normal cells into cancer cells [2]. Various types of CIN have been detected in glioma, including mutations, loss of heterozygosity, and copy number aberrations [3–6]. Several studies have reported that CIN can affect sensitivity to chemotherapy and consequently the prognosis of glioma patients [7, 8].

A close association between CIN and histone acetylation has been demonstrated [9–11]. Central to histone acetylation are histone deacetylases (HDACs), which maintain genomic integrity by targeting histone and non-histone proteins and thereby regulating DNA repair mechanisms [12]. A total of 18 human HDACs, classified into four groups, have been identified. As a member of group II HDACs, HDAC4 is closely linked to many disease processes—including cancer, leukemia, diabetes, infection, and cardiac disease [13–18]—and is also highly expressed in the brain where it plays an important role in brain functioning [19–22].

Epigenetic silencing of the *O*-*6*-*methylguanine*-*DNA methyltransferase* (*MGMT*) gene by promoter methylation is associated with prolonged survival and sensitivity to chemotherapeutic alkylating agents in GBM patients undergoing standard treatment [23, 24]. The beneficial effects of combined radiochemotherapy vary significantly between GBM patients, even for those with a methylated *MGMT* promoter [25]. This suggests that while important, *MGMT* promoter methylation is not the sole factor determining clinical outcome, and highlights the need for evaluating patients based on other factors; for instance, CIN combined with *MGMT* promoter methylation status may provide more accurate information for predicting disease outcome.

CIN is defined as the gain or loss of whole or fractions of chromosomes, and is associated with tumorigenesis, disease prognosis, and acquisition of multi-drug resistance in various cancers, including breast cancer, melanoma, and lymphoma [26–32]. High throughput gene expression profiling approaches have established a reasonable link between the expression of specific genes and the degree of CIN in multiple cancers. Carter et al. developed computational methods to measure the “CIN score” for 10,151 genes, which indicates the correlation between each gene and the CIN degree in tumor samples [30]. Based on the “CIN score”, the top ranked genes are chosen for forming the CIN signature, which was represented by CIN25 score (Further backgrounds of CIN signature and CIN25 score are shown in the Supplementary Text) [30]. The CIN signature, comprising a specific set of genes that are critical for maintaining genomic integrity, is significantly higher in metastatic foci, and stratifies patients according to clinical outcome in various cancers, suggesting that these genes are responsible for a more aggressive cancer phenotype [30, 33]. However, as it consists of multiple genes, the CIN signature is too complex to be suitable for routine clinical application. The present study investigated whether *HDAC4* expression can serve as an alternative marker for assessing the degree of CIN and, in combination with *MGMT* promoter status, predict the outcome of patients.

## Materials and methods

### Patients and samples

A total of 539 glioma specimens from the Chinese Glioma Genome Atlas (CGGA) that were contiguously collected at multiple centers were used in this study. Tumor tissue samples were obtained by surgical resection prior to radio and/or chemotherapy, flash-frozen in liquid nitrogen, and stored at −80 °C until nucleic acid extraction. The study protocol was approved by the ethics committees of participating hospitals. Each sample was diagnosed and independently confirmed histopathologically at the Department of Pathology according to the 2007 WHO classification system of CNS tumors by two experienced neuropathologists. Clinical data, including age, sex, preoperative KPS score, adjuvant radiation and chemotherapy, and the recorded date of disease progression or death were obtained from medical records.

Data on mRNA expression were obtained by whole transcriptome sequencing (N = 325) and whole-genome mRNA expression microarray (N = 299) from the CGGA, and the following four datasets were used for validation: the Cancer Genome Atlas (http://cancergenome.nih.gov); Repository for Molecular Brain Neoplasis Data (REMBRANDT, http://caintegrator.nci.nih.gov/rembrandt); GSE16011 (http://www.ncbi.nlm.nih.gov/geo/query/acc.cgi?acc=GSE16011); and GSE4290 (http://www.ncbi.nlm.nih.gov/geo/query/acc.cgi?acc=GSE4290).

### Evaluation of MGMT promoter methylation by DNA pyrosequencing

*MGMT* promoter methylation status was detected by DNA pyrosequencing as previously described [34, 35]. Bisulfite DNA modification was performed using the EpiTect Kit (Qiagen). The following primers were used to amplify the *MGMT* promoter region: 5′-GTTTYGGATATGTTGGGATA-3′ (forward) and 5′-biotin-ACCCAAACACTCACCAAATC-3′ (reverse). The PCR analysis was performed in duplicate in a 40-μl reaction volume containing 0.5 μl each primer (using a 10-μM working solution), 4 μl 10 × buffer, 3.2 μl of 2.5 μM dNTP, 2.5 U hotstart *Taq* (Takara Bio, Madison, WI, USA), and 2 μl of 10 μM bisulphite-treated DNA. The reaction conditions were: 95 °C for 3 min; 40 cycles of 95 °C for 15 s, 52 °C for 30 s, and 72 °C for 30 s; and 72 °C for 5 min (ABI 9700; Applied Biosystems, Foster City, CA, USA). DNA was purified from total PCR products using QIAamp DNA Mini Kit (Qiagen, Valencia, CA, USA) and subjected to pyrosequencing (PyroMark Q96 ID System; Qiagen) using the primer 5′-GGATATGTTGGGATAGT-3′ in accordance with the manufacturer’s instructions. The obtained methylation values were averaged across the seven tested CpG loci within the *MGMT* promoter. Samples were considered as having a methylated *MGMT* promoter if the average methylation was ≥10 %.

### Survival analysis

To assess the prognostic value of *HDAC4* expression in glioma, a survival analysis for each tumor grade was performed based on expression level. The combined effect of *MGMT* promoter methylation status and *HDAC4* expression was then assessed in GBM. Patients receiving radiochemotherapy were stratified into two groups according to *MGMT* status, and further classified into four subgroups based on *HDAC4* expression level; the statistical significance was determined by the log-rank test.

CIN25 score based on 25 genes was calculated as the sum of the expression levels of each signature gene in a patient [30]. Dichotomization was performed for each tumor grade to classify patients into two groups based on the median signature score. Patients who received radiochemotherapy were then stratified into four subgroups based on *MGMT* status and CIN25 score to study the combined effect of these parameters in GBM, and a survival analysis was carried out.

### Gene ontology (GO) analysis and gene set enrichment analysis (GSEA)

A Pearson correlation analysis was performed across glioma grades to identify genes that are significantly related to *HDAC4*. GO analysis was performed using the DAVID (http://david.abcc.ncifcrf.gov/home.jsp) [36]. To obtain more information about the relationship between CIN25 score and *HDAC4*, GSEA (http://www.broadinstitute.org/gsea/index.jsp) was performed as previously described to determine whether the identified set of genes showed statistically significant differences between the two biological states [37].

### Statistical analysis

SPSS software and GraphPad Prism 6 were used for statistical analyses. The differences in *HDAC4* expression and CIN25 score between groups were compared using Student’s t and χ^2^ tests. A dichotomization based on the median *HDAC4* expression level and CIN25 score was carried out for the survival analysis. Overall survival (OS) was calculated from the date of diagnosis until death or the end of follow-up. Progression-free survival (PFS) was defined as the time between the diagnosis and the first unequivocal clinical or radiological sign of disease progress. Kaplan–Meier survival analyses for OS and PFS were performed and compared with the log-rank test. A Pearson correlation analysis was used to test the correlation between CIN25 score and *HDAC4* expression. Statistical significance was defined as a two-tailed P value < 0.05.

## Results

### HDAC4 expression is significantly associated with progressive malignancy in glioma

**Fig. 1:**
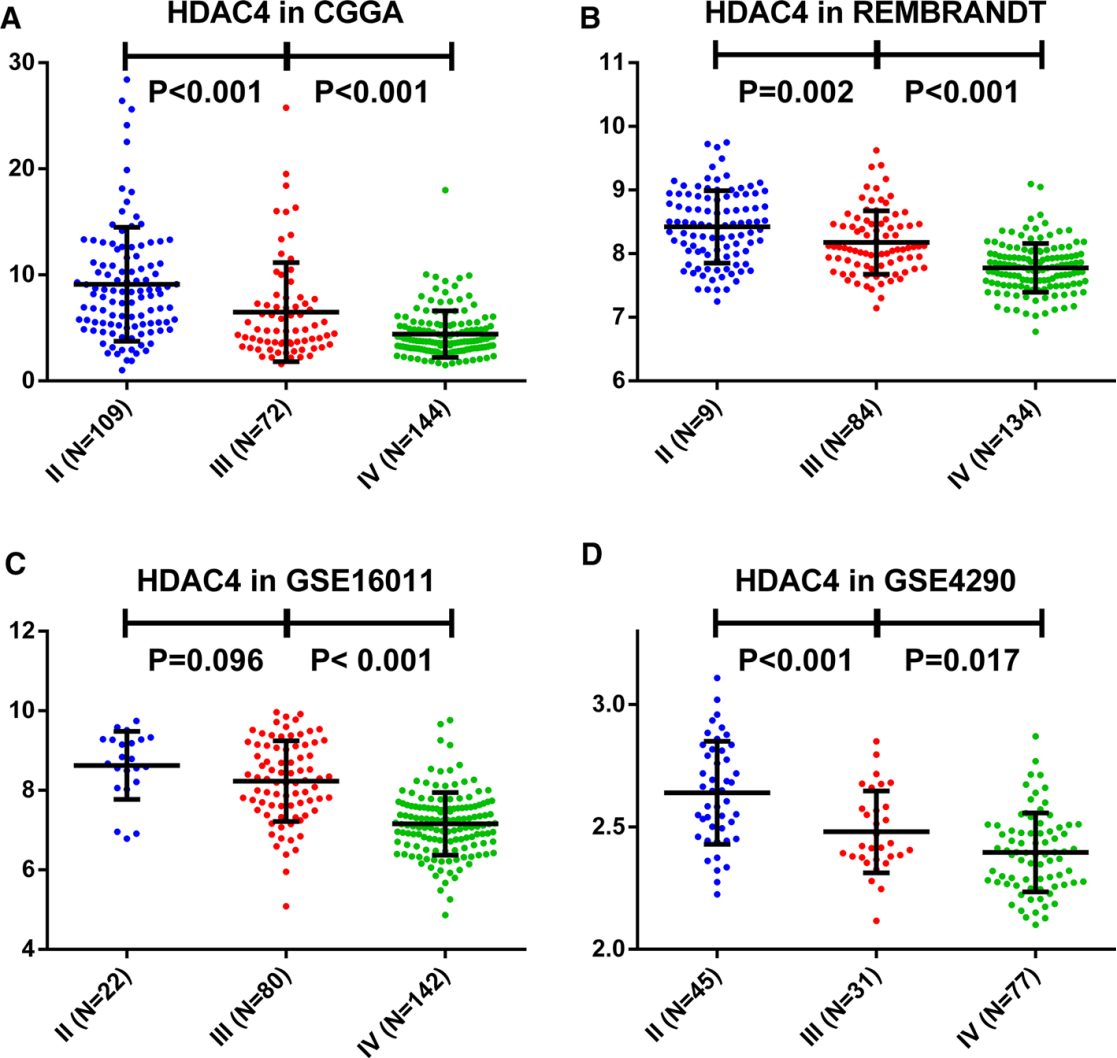
*HDAC4* expression is negatively correlated with tumor grade. The association between *HDAC4* expression level and grade II, III, and IV glioma was evaluated in the CGGA (**a**) and three other validation sets (**b**–**d**)

To test the relationship between *HDAC4* expression and tumor grade, patients were stratified into low or high expression groups according to the median value for *HDAC4* expression in each database. The percentage of samples with low expression increased with progressive malignancy (P < 0.001; χ^2^ test) (Table S1). This correlation between *HDAC4* expression and tumor grade was studied in CGGA and three validation sets (Fig. 1a–d) showing that *HDAC4* expression differed among various grades and was downregulated for higher grades. Based on these results, we propose that low *HDAC4* expression is a characteristic of high-grade glioma.

### Highly expressed HDAC4 prolongs survival and strengthens the predictive value of MGMT promoter methylation

**Fig. 2:**
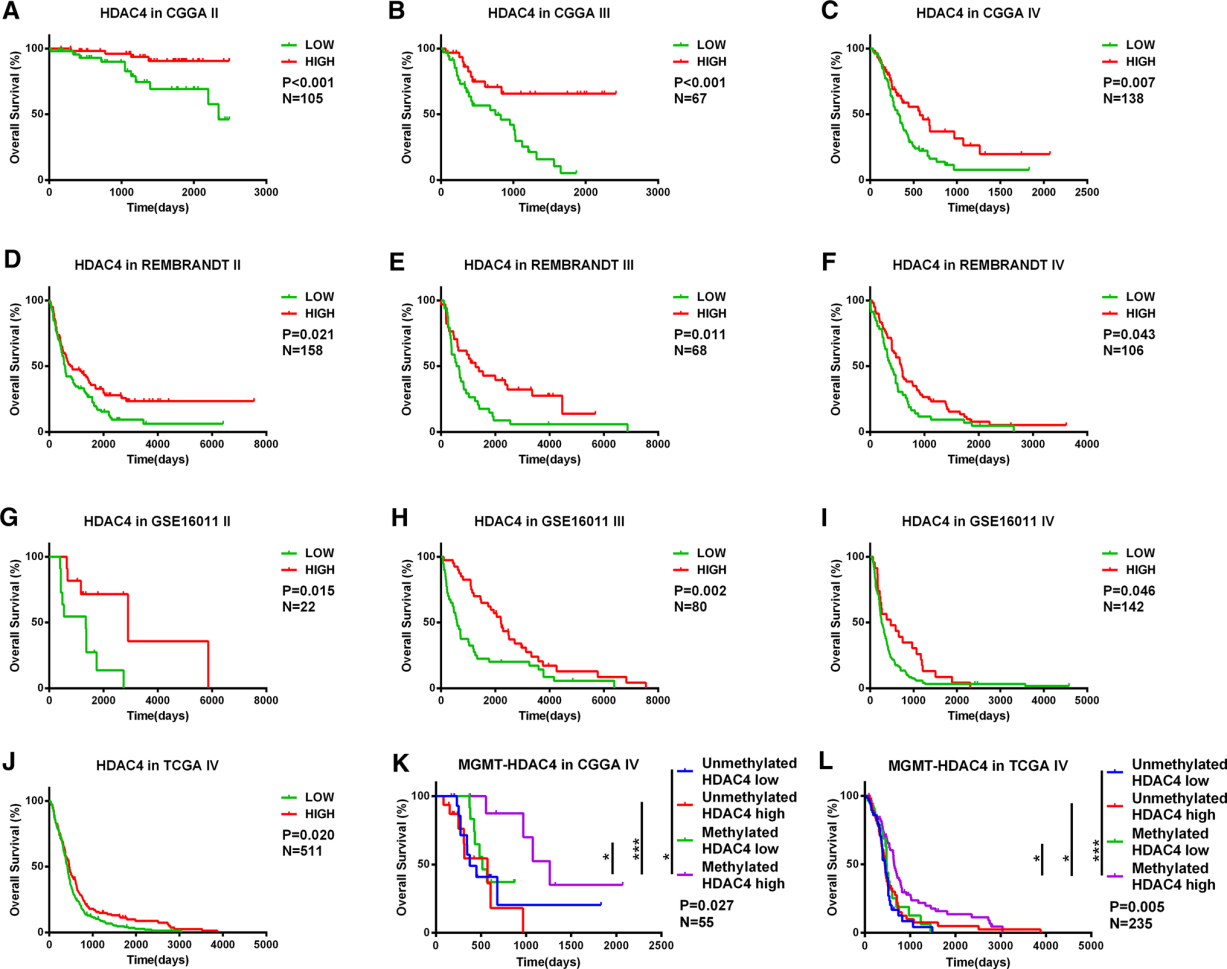
Higher *HDAC4* expression is associated with longer OS in the CGGA, REMBRANDT, GSE16011, and TCGA databases (**a**–**j**). Survival analysis according to *MGMT* promoter status combined with *HDAC4* expression was performed with data from the CGGA (**k**) and TCGA (**l**). Patients whose tumors had a methylated *MGMT* promoter and a higher expression of *HDAC4* had the best prognosis

To evaluate the prognostic value of *HDAC4* expression in glioma, dichotomization was applied in every grade to separate samples into two groups based on *HDAC4* expression level. Kaplan–Meier survival curves in CGGA and three validation sets showed that *HDAC4* overexpression conferred longer OS (Fig. 2a–j) and PFS (Fig. S1a–c) in each grade. When both radiochemotherapy and *MGMT* promoter methylation status were considered, 55 GBM patients were included in the assessment of the prognostic value of *HDAC4* expression combined with *MGMT* promoter methylation status. Patients were assigned to four subgroups according to their *MGMT* status and *HDAC4* expression level, as described above. Notably, OS varied significantly among the four subgroups (P = 0.027; Fig. 2k). Among patients with a methylated *MGMT* promoter, those with higher *HDAC4* expression had a median OS of 669 days, which was significantly longer than that of patients with low *HDAC4* expression or with a non-methylated *MGMT* promoter (Fig. 2k). There were no other differences among the three subgroups. A similar analysis was carried out in GBM patients who had received standard radiation combined chemotherapy in the TCGA database, and the results confirmed that patients with *MGMT* promoter methylation and high *HDAC4* expression had a significantly longer OS than other patients (Fig. 2l).

### HDAC4 expression is closely correlated with chromatin structure

A Pearson correlation analysis was conducted to identify genes whose expression is correlated with that of *HDAC4*. A total of 4,794 genes were significantly correlated (3,262 genes with R < − 0.3 and 1,532 genes with R > 0.3; P < 0.001). Positively correlated genes with a P value < 1e^−10^ were used for the GO analysis, which revealed ten processes mostly related to chromatin organization and histone modification. These results confirm that *HDAC4* is critical for regulating chromosome structure (Table 1).

**Table 1:** Gene ontology (GO) terms for *HDAC4*-associated genes

Term	Count	P value	Fold enrichment
GO:0006325 ~ chromatin organization	27	0.04587	1.47075
GO:0016568 ~ chromatin modification	21	0.04497	1.57811
GO:0016569 ~ covalent chromatin modification	14	0.00810	2.28784
GO:0016570 ~ histone modification	14	0.00621	2.36285
GO:0016573 ~ histone acetylation	10	0.00043	4.28970
GO:0043966 ~ histone H3 acetylation	7	0.00127	5.54361
GO:0043983 ~ histone H4-K12 acetylation	3	0.03092	10.29528
GO:0043982 ~ histone H4-K8 acetylation	3	0.03092	10.29528
GO:0043984 ~ histone H4-K16 acetylation	3	0.03092	10.29528
GO:0043981 ~ histone H4-K5 acetylation	3	0.03092	10.29528

### A high CIN25 score is associated with progressive malignancy, poor prognosis, and chemotherapy resistance in glioma

**Fig. 3:**
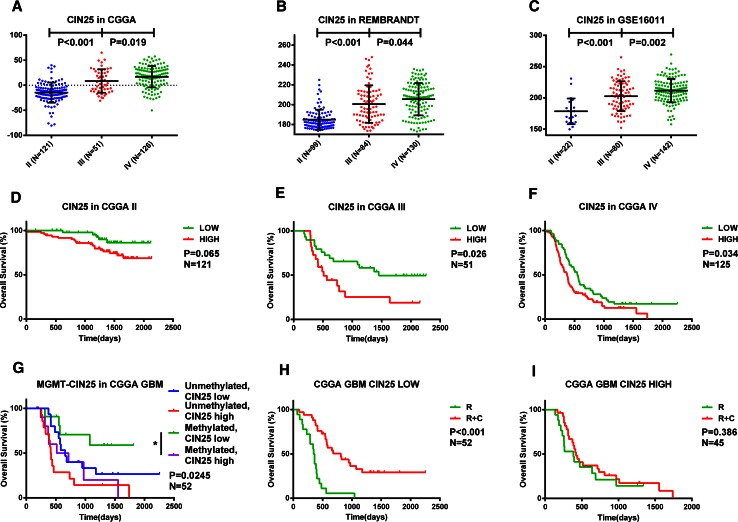
Correlation between CIN25 score and glioma malignancy. CIN, as measured by the CIN25 score, was analyzed with respect to tumor grade (II–IV) in the CGGA (**a**) and two validation sets (**b**, **c**). A high CIN25 score was associated with shorter OS (**d**–**f**) in the CGGA. A survival analysis for *MGMT* promoter status combined with CIN25 score was performed in the CGGA (**g**). Sensitivity to chemotherapy was assessed in GBM patients. Patients with a low CIN25 score receiving radiochemotherapy had better OS than those receiving radiotherapy alone (**h**); no differences between treatment groups were observed among patients with a high CIN25 score (**i**)

The CIN25 score was used as a marker of CIN. Based on the median score across tumor grades, patients were stratified into low and high score groups. Patients with high scores has greater representation among higher tumor grades (P < 0.001; χ^2^ test) (Table S2). Analysis of data from the CGGA and two validation databases (Student’s t test) (Fig. 3a–c) showed that CIN25 scores increased as a function of glioma grade and was highest in the most malignant GBM, indicating that CIN is tightly associated with glioma progression.

The prognostic value of the CIN25 score in glioma was next evaluated across three independent databases. The OS and PFS showed a notable reduction for patients with a high as compared to a low CIN25 score (Fig. 3d–f, Fig. S2, S3). In 52 GBM patients who received post-operative radiochemotherapy, *MGMT* promoter methylation status and CIN25 score were incorporated into the stratification; in this case, the OS differed significantly among subgroups (P = 0.025; Fig. 3g). Patients with *MGMT* promoter methylation and lower CIN25 score had a longer OS than the other three subgroups. A significant difference in clinical outcome was detected in the *MGMT* promoter methylation group, demonstrating that a low CIN25 score was associated with better prognosis than a high CIN25 score (P = 0.046; Fig. 3g). When the relationship between CIN25 score and response to chemotherapy in GBM was analyzed, patients with a lower CIN25 score who received radiochemotherapy had longer survival times than those who received radiotherapy alone (P < 0.001, Fig. 3h). However, there was no difference between these two treatment conditions among patients with high CIN25 scores (Fig. 3i), suggesting that the poor prognosis observed in these patients may be due to the acquisition of chemotherapy resistance.

### CIN signature is strongly associated with HDAC4 expression in glioma

**Fig. 4:**
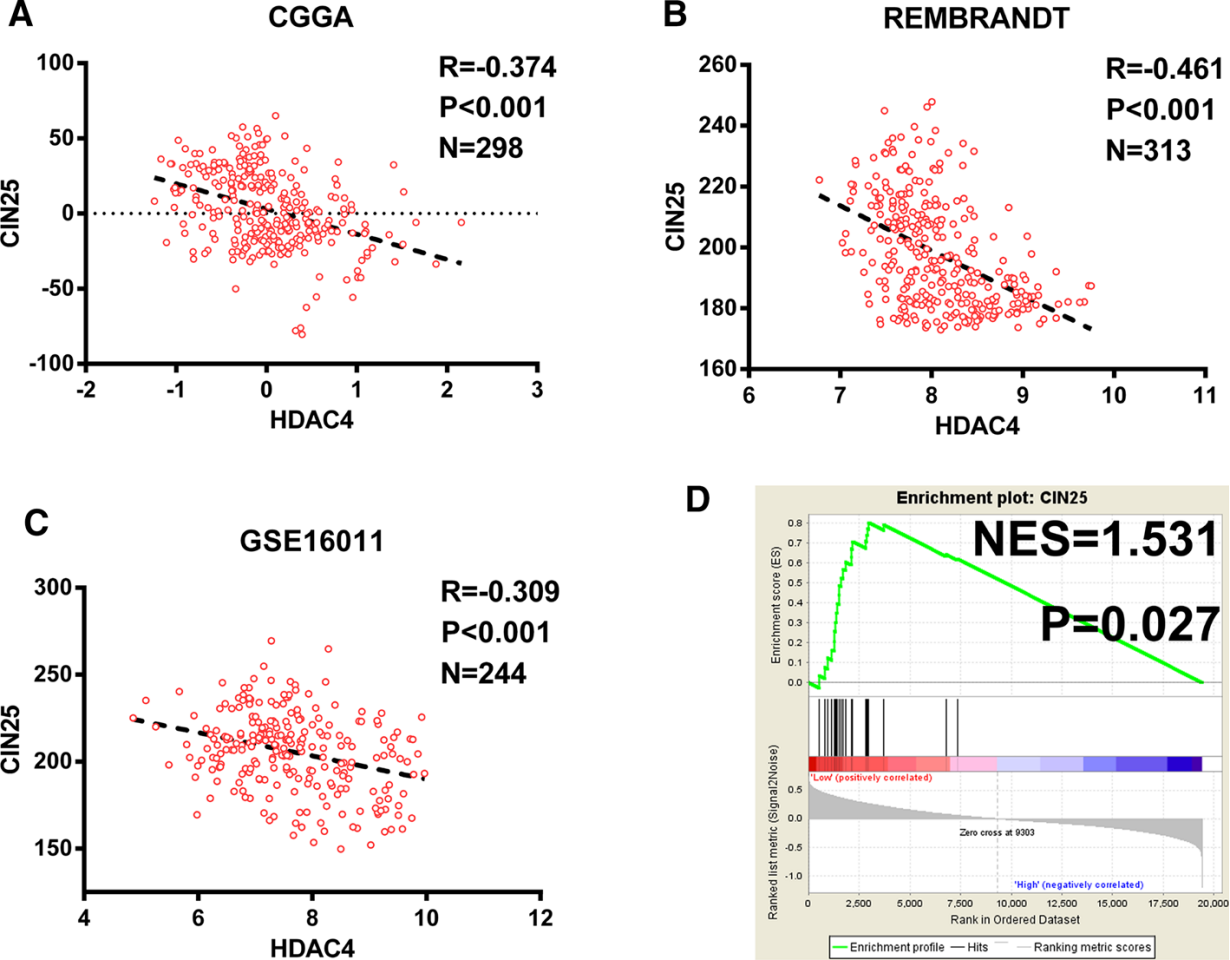
Association between *HDAC4* expression and *CIN25* in glioma. *HDAC4* expression level was closely correlated with CIN25 score in the CGGA (**a**) and other validation sets (**b**, **c**). The GSEA showed that CIN25 genes were significantly enriched in samples with low *HDAC4* expression (**d**). The *horizontal bar* in graded color from *red* to *blue* represents the rank ordering of patients based on increasing *HDAC4* expression. The *vertical black lines* represent the projection of individual genes constituting the CIN25 score. Genes on the left (*red*) correlated most strongly with downregulated *HDAC4* expression. *NES* normalized enrichment score

A correlation analysis revealed that *HDAC4* expression was significantly correlated with CIN25 score (Fig. 4a, P < 0.001, R = − 0.366), which was confirmed by data from the REMBRANDT and GSE16011 datasets (P < 0.001, R = − 0.461 and P < 0.001, R = − 0.309, respectively; Fig. 4b, c). This suggests a significant relationship between *HDAC4* expression and CIN in glioma. The GSEA was used to test whether *HDAC4* expression is correlated with genes contributing to the CIN25 score. Samples were listed in order of increasing *HDAC4* expression. The results indicate that CIN25 genes were significantly enriched in samples with low *HDAC4* expression, whereas high *HDAC4* expression was not correlated with any of these genes (NES = 1.5306281, P = 0.027; Fig. 4d).

## Discussion

The high prevalence, mortality, and risk of post-treatment complications associated with glioma make it one of most challenging diseases affecting humans. Moreover, patients with the same diagnosis may experience vastly different clinical outcomes even after undergoing the same treatment. This heterogeneity highlights the limitations of a grading system based purely on pathological characterization. As a feature of most human cancers, CIN—which has a high degree of heterogeneity among tumor cells and involves a complex network of molecular interactions rather than a single signaling pathway [38]—may better reflect glioma severity and offer a more accurate measure for predicting disease prognosis.

HDACs are the main regulators of histone acetylation, which has been implicated in CIN. As a member of group II HDACs, *HDAC4* is highly expressed in the brain and involved in various functions including learning and memory, behavior, and neuronal survival [19–21, 39, 40]. We propose that *HDAC4* expression is closely associated with glioma grade and prognosis, with a lower *HDAC4* expression significantly associated with progressive malignancy and unfavorable disease outcome, similar to what is observed in other cancers [41]. The GO analysis indicated that *HDAC4* expression is functionally related to the maintenance of chromosome structure. Meanwhile, several previous studies have demonstrated various roles for *HDAC4* in cancer cells. In chondrosarcoma, a decrease in *HDAC4* expression leads to the upregulation of vascular endothelial growth factor expression, thereby stimulating angiogenesis [42]; in prostate cancer cells, *HDAC4* downregulation was associated with a high level of androgen receptor expression, which promoted cell growth [43]. The results of the present study reveal that *HDAC4* is a strong prognostic factor in glioma and likely determines patient outcome via modulation of genomic integrity.

The most widely used marker of genomic instability is the CIN signature, which embodies aberrations in chromosome number as well as structure [44]. There was considerable overlap in the CIN25 score across tumor grades, and patients with the same grade had significantly different CIN25 scores. The overlap between grades was normalized by restricting the analysis of CIN25 score to each grade in the glioma databases; as in the case of other cancers, a higher score was linked to high pathological grade and unfavorable prognosis, confirming that a loss of genomic integrity plays an important role in tumorigenesis and impacts patient prognosis. The strongly negative correlation between *HDAC4* expression and CIN which determined by statistical analyses of GO results and by GSEA confirmed the utility of *HDAC4* as a more convenient, alternative marker of genomic instability.

Previous studies have reported that cancer cells with a high degree of CIN acquire multi-drug resistance at higher rates as compared to diploid cells with stable chromosomes. This is true in the case of colorectal cancer, regardless of somatic mutation status [45]. A similar relationship to drug sensitivity was found in soft tissue sarcoma and ovarian cancer [32, 46]. In this study, GBM patients with a low CIN25 score were more sensitive to radiochemotherapy and lived longer than those receiving radiotherapy alone, but there were no differences observed between the two treatment groups for patients with a high CIN25 score. The relationship between CIN and multi-drug resistance can be explained by the increased heterogeneity in malignant cancers resulting from CIN, which increases the probability of a drug-resistant subclone arising in the tumor [47].

Combined radiochemotherapy, rather than radiotherapy or chemotherapy alone, is a standard treatment for GBM. *MGMT* promoter methylation status is a clinical predictor of the extent to which GBM patients will benefit from chemotherapy [25]. Several studies have shown that *MGMT* deficiency resulting from *MGMT* promoter methylation may confer increased sensitivity to alkylating agents, yet some glioma patients with *MGMT* promoter methylation still exhibit resistance to these drugs [25, 48]. Thus, evaluating genomic integrity in combination with *MGMT* promoter methylation status may provide additional insight into the mechanism underlying the acquisition of drug resistance [49]. The current analysis of GBM patients with *MGMT* promoter methylation receiving combined radiochemotherapy revealed that survival was prolonged in patients with a low CIN25 score (indicating a more stable genome) than those with a high score; in the latter group, the poor outcome was likely due in part to chemotherapy resistance arising from increased CIN. These results indicate that the sensitivity to chemotherapy conferred by *MGMT* methylation depends on a stable genome, and that the degree of genomic instability further stratifies patients with *MGMT* methylation. The CIN25 score encompasses the status of 25 different genes, and as such, is difficult to incorporate into routine clinical practice as a diagnostic tool. Based on the strong association between *HDAC4* expression and CIN, we examined whether the combination of *MGMT* promoter methylation status and *HDAC4* expression level could instead be used to predict patient outcome. Interestingly, for the highly malignant GBM, combined radiochemotherapy had the greatest benefit for patients with *MGMT* promoter methylation and high *HDAC4* expression (indicating a lesser degree of CIN). Thus, these two factors combined can identify patients with the best prognosis who are suitable candidates for more aggressive therapy, even the underlying mechanisms needed further experimental methods for interpretation.

Such an analysis helped us gain a novel perspective for understanding the chemotherapy resistance in GBM patients. The mechanisms of chemotherapy resistance resulted from CIN needed further experimental methods for interpretation. In addition, whether the CIN degree was a determining factor in gliomagenesis of different subtypes and further tightly associated with the appearance of MGMT promoter methylation in GBM patients were pertinent questions as well. With immunohistochemistry being widely used in both routine clinical practice and research, the role of *HDAC4* in glioma could be further validated from protein level in the near future. Clinically, our next major goal is to verify its role in the guidance of glioma diagnosis and treatment.

In conclusion, in present study, *HDAC4* expression was found to be closely related to tumor grade and patient prognosis, and functional and statistical analyses identified a correlation between *HDAC4* expression and CIN signature in glioma. Taken together, the results indicate that *HDAC4* can serve as a marker of CIN and, when combined with *MGMT* promoter methylation status, may be used to identify GBM patients who would benefit most from combined radiochemotherapy.

## Electronic supplementary material

Below is the link to the electronic supplementary material.

Supplementary material 1 (DOC 35 kb)

Supplementary material 2 (TIFF 192 kb)

Supplementary material 3 (TIFF 194 kb)

Supplementary material 4 (TIFF 146 kb)

Supplementary material 5 (DOCX 13 kb)
