# Supplementary material for: HDAC4, a prognostic and chromosomal instability marker, refines the predictive value of MGMT promoter methylation
Source: J Neurooncol. 2015 Jan 4;122(2):303–12. doi: 10.1007/s11060-014-1709-6 (PMC4368847; doi:10.1007/s11060-014-1709-6)
Supplement: Supplementary file 1 — Supplementary material 1 (DOC 35 kb) [file 11060_2014_1709_MOESM1_ESM.doc]

**Supplementary text**

CIN is one of the most consistent characteristics of human cancers . Carter et al. developed a computational method to measure CIN in tumor samples based on the expression of specific genes that are consistently correlated with CIN in several tumor types, such as glioma, breast cancer, lung cancer, and lymphoma. In their paper, 10,151 genes were ranked according to a specific score that represented the expression level of each gene with respect to the degree of CIN in multiple cancer types. The top 25 genes that were most relevant to CIN constituted the CIN25. The specific set of genes involved in cellular processes critical for maintenance of genetic integrity that were relevant both clinically and biologically. The clinical outcome across multiple cancer types could be stratified by the CIN25 score, indicating the importance of these genes in identifying an aggressive cancer phenotype. On the other hand, these 25 genes encode members of one or more functional modules that are associated with or cause CIN when overexpressed. The CIN25 score for a given sample was calculated as the sum of the expression levels of these 25 genes, further proposing a measure for assessing CIN degree by a given set of genes. Genes formed the CIN25 score were listed in the table below

| **Genes of CIN25 score** | | | | |
| --- | --- | --- | --- | --- |
| CCNB2 | CCT5 | CDC2 | CDC45L | ESPL1 |
| FEN1 | FOXM1 | H2AFZ | KIF20A | MAD2L1 |
| MCM2 | MCM7 | MELK | NCAPD2 | PCNA |
| PRC1 | RAD51AP1 | RFC4 | RNASEH2A | TGIF2 |
| TOP2A | TPX2 | TRIP13 | TTK | UBE2C |
