# Supplementary material for: HDAC4, a prognostic and chromosomal instability marker, refines the predictive value of MGMT promoter methylation
Source: J Neurooncol. 2015 Jan 4;122(2):303–12. doi: 10.1007/s11060-014-1709-6 (PMC4368847; doi:10.1007/s11060-014-1709-6)
Supplement: Supplementary file 5 — Supplementary material 5 (DOCX 13 kb) [file 11060_2014_1709_MOESM5_ESM.docx]

**Figure S1.**

A higher *HDAC4* expression is associated with longer PFS in the CGGA database.

**Figure S2.**

A higher CIN25 score is correlated with shorter PFS in the CGGA database.

**Figure S3.**

A higher CIN25 score is correlated with poor OS in two validation sets (REMBRANDT and GSE16011).

**Table S1. Distribution of *HDAC4* in databases**

|  | **Grade II** | **Grade III** | **Grade IV** | **P value** |
| --- | --- | --- | --- | --- |
| **CGGA Database** |  |  |  |  |
| HDAC4-Low (N = 162) | 22 (20.2%) | 38 (52.8%) | 102 (70.8%) | < 0.001 |
| HDAC4-High (N = 163) | 87 (79.8%) | 34 (47.2%) | 42 (29.2%) |  |
| **REMBRANDT Database** |  |  |  |  |
| HDAC4-Low (N = 156) | 24 (24.2%) | 32 (38.1%) | 100 (76.9%) | < 0.001 |
| HDAC4-High (N = 157) | 75 (75.8%) | 52 (61.9%) | 30 (23.1%) |  |
| **GSE16011 Database** |  |  |  |  |
| HDAC4-Low (N = 122) | 3 (13.6%) | 19 (23.8%) | 100 (70.4%) | < 0.001 |
| HDAC4-High (N = 122) | 19 (86.4%) | 61 (76.2%) | 42 (29.6%) |  |
| **GSE4290 Database** |  |  |  |  |
| HDAC4-Low (N = 76) | 10 (22.2%) | 17 (54.8%) | 49 (63.6%) | < 0.001 |
| HDAC4-High (N = 76) | 35 (77.8%) | 14 (45.2%) | 28 (36.4%) |  |

**Table S2. Distribution of CIN25 in databases**

|  | **Grade II** | **Grade III** | **Grade IV** | **P value** |
| --- | --- | --- | --- | --- |
| **CGGA Database** |  |  |  |  |
| CIN25-Low (N = 149) | 100 (82.6%) | 23 (45.1%) | 26 (20.6%) | < 0.001 |
| CIN25-High (N = 149) | 21 (17.4%) | 28 (54.9%) | 100 (79.4%) |  |
| **REMBRANDT Database** |  |  |  |  |
| CIN25-Low (N = 156) | 85 (85.9%) | 38 (45.2%) | 33 (25.4%) | <0 .001 |
| CIN25-High (N = 157) | 14 (14.1%) | 46 (54.8%) | 97 (74.6%) |  |
| **GSE16011 Database** |  |  |  |  |
| CIN25-Low (N = 122) | 20 (90.9%) | 46 (57.5%) | 56 (39.4%) | < 0.001 |
| CIN25-High (N = 122) | 2 (9.1%) | 34 (42.5%) | 86 (60.6%) |  |
